# Effect of nutrient supplementation on the acquisition of humoral immunity to *Plasmodium falciparum* in young Malawian children

**DOI:** 10.1186/s12936-018-2224-6

**Published:** 2018-02-07

**Authors:** Priyanka Barua, Upeksha P. Chandrasiri, James G. Beeson, Kathryn G. Dewey, Kenneth Maleta, Per Ashorn, Stephen J. Rogerson

**Affiliations:** 1 Department of Medicine (RMH), Peter Doherty Institute for Infection and Immunity, University of Melbourne, Melbourne, VIC Australia; 2 Burnet Institute for Medical Research and Public Health, Melbourne, VIC Australia; 3 Monash University, Melbourne, VIC Australia; 4 University of California, Davis, CA USA; 5 University of Malawi, Zomba, Malawi; 6 University of Tampere and Tampere University Hospital, Tampere, Finland

**Keywords:** Malarial immunity in children, Nutrient supplements, Randomized controlled trial, Merozoite antigens, Variant surface antigens, Seroprevalence

## Abstract

**Background:**

There is evidence that suggests that undernutrition has a detrimental effect on malarial immunity in children. The aim of the study was to discover whether nutrient supplementation improved development of malarial antibody immunity in children up to 18 months of age.

**Methods:**

The study was conducted with a subset of 432 Malawian children from a randomized controlled trial of nutritional supplements. The arms included pre- and postnatal small-quantity lipid-based nutrient supplements for both mother and child; prenatal supplementation with iron and folic acid; and pre- and postnatal supplementation with multiple micronutrients. Paired plasma samples were collected at 6 and 18 months of age. The levels of antibodies against merozoite surface protein 1 (MSP1 19kD) and MSP2, erythrocyte binding antigen 175 (EBA175), reticulocyte binding protein homologue 2A (Rh2A9), schizont extract and variant antigens expressed on the surface of infected erythrocytes were measured.

**Results:**

At 18 months of age, 5.4% of children were parasitaemic by microscopy and 49.1% were anaemic. Antibodies to the tested merozoite antigens and schizont extract increased between 6 and 18 months and this increase was statistically significant for MSP1, MSP2 and EBA175 (p < 0.0001) whereas IgG to variant surface antigens decreased with increasing age (p < 0.0001). However, the supplementation type did not have any impact on the prevalence or levels of antibodies at either 6 or 18 months of age to any of the tested malaria antigens in either univariate analysis or multivariate analysis after adjusting for covariates.

**Conclusions:**

Pre- and postnatal lipid-based nutrient supplementation did not alter malaria antibody acquisition during infancy, compared to prenatal supplementation with iron and folic acid or pre- and postnatal supplementation with multiple micronutrients.

*Trail registeration* Clinicaltrials.gov registration number NCT01239693

## Background

Malaria is one of the leading causes of death in children and pregnant women with an estimated 214 million new cases and 438,000 deaths worldwide in 2015. The disease can be caused by five different species of the genus *Plasmodium*, of which *Plasmodium falciparum* causes the highest rates of mortality and morbidity and is particularly prominent in young children of sub-Saharan Africa, with an estimated 292,000 deaths in 2015 [1].

In sub-Saharan Africa, malaria and malnutrition often co-exist, and both contribute significantly to deaths in young children. However, studies of possible synergistic clinical effects of malaria and malnutrition have given conflicting results, indicating the need for further studies in this area. For example, in a cross-sectional study among pre-school Kenyan children [2] and a longitudinal study in Gambian children under 5 years of age [3], stunting was associated with increased malarial risk, but in Papua New Guinea it was reported that stunting might protect children against clinical malaria episodes [4]. Some other studies noted no significant association between anthropometric measurements [5], stunting [6] or undernutrition [7] and altered susceptibility to malaria.

A limited number of studies have examined the impact of nutrient supplementation on malaria susceptibility in children. Zinc and vitamin A supplementation reduced clinical malaria episodes caused by *P. falciparum* in young children [8–10]. In a high malaria transmission setting, iron supplementation was associated with increased parasitaemia [11] and increased mortality [12] in iron-sufficient children, whereas the provision of iron with micronutrients was associated with reduced risk of malaria in iron-deficient children [13]. Other studies have found evidence of associations between acute malaria and deficiency of thiamine [14] and antioxidants including vitamin E [15], which suggests they have roles in protection against malaria. While there is limited evidence that supplementation with micronutrients such as zinc or vitamin B12 can improve antibody response to vaccination [16, 17], the ability of micro- or macronutrient supplementation to affect the acquisition of antibody to pathogens following natural exposure is unknown.

The aim of this study was to identify whether pre- and postnatal nutritional supplements could improve malarial immunity in young children. The study was part of a nutrient supplementation clinical trial, the International Lipid-based Nutrient Supplement (iLiNS) Project DYAD-Malawi trial (clinicaltrials.gov registration number NCT01239693). For this report, the level and prevalence of antibody to merozoite antigens, schizont extract and variant surface antigens (VSA) expressed by *P. falciparum*-infected erythrocytes (IEs) were determined in infants aged 6 and 18 months as antibodies to merozoite antigens and VSAs are believed to play important roles in mediating acquired immunity against malaria [18, 19].

## Methods

### Study location and participants

The study participants were a cohort of 432 infants residing in Lungwena, Malindi and Mangochi from rural Malawi who participated in the iLiNS Project DYAD-Malawi nutrient supplementation trial, part of the iLiNS Project [20]. The details of the trial design and supplements have been published elsewhere [21]. In brief, participating pregnant women were randomly allocated to receive iron and folic acid (IFA), multiple micronutrients (MMN) or a small-quantity (20 g) of lipid based nutrient supplement (LNS) daily. After delivery, women in the IFA group received placebo tablets, while MMN and LNS supplementation was continued during the first 6 months of lactation. Children of mothers in the LNS group also received LNS 10 g twice daily from 6 to 18 months of age. At 18 months of age, anthropometric assessments revealed no significant differences in the children’s mean length, mean weight, the prevalence of stunting, or head or mid-upper arm circumference between the intervention groups for all participants in the iLiNS project [22]. Plasma samples were collected from infants at 6 and 18 months of age. At these time points blood haemoglobin concentration was measured with a Hemo-Cue^®^ haemoglobinometer from venous blood samples. Malaria parasitaemia was sought by microscopic examination of thick blood film and by rapid diagnostic test (RDT) using Clearview^®^ Malaria Combo (British Biocell International Ltd., Dundee, UK).

### Plasma samples preparation

Blood from participants was separated by centrifugation shortly after collection and plasma was stored at − 80 °C before shipment on dry ice to Australia. Plasma samples were heat-inactivated for 45 min at 57 °C to inactivate complement proteins. The heat-inactivated samples were then stored at − 80 °C.

### Culturing and maintaining parasites

The *P. falciparum* lines used were E8B-ICAM, R29 and 3D7 *var*A over-expressing parasite line. E8B-ICAM adheres to ICAM-1 and CD36 [23], and expresses group B/C *var* genes whereas R29 expresses group A *var* genes and forms rosettes [24]. The 3D7 line spontaneously expressed a group A *var* gene as its dominant transcript [25]; its binding ligands have not been characterized. The parasites were grown and maintained in culture as described previously [26]. IEs were synchronized with 5% sorbitol and subject to gelatin flotation regularly [27]. To select R29 for rosetting, gelatin flotation without then with heparin lithium salt, 0.05 mg/ml Sigma Aldrich), was performed.

### Measuring IgG to malaria merozoite antigens and schizont extract

Recombinant merozoite protein 1 (MSP-1 19 kD, 3D7 clone), region III-V of erythrocyte binding antigen 175 (EBA 175), and *P. falciparum* reticulocyte binding homologue 2 (PfRh2, construct PfRh2-2030) were expressed in *Escherichia coli* as previously reported [28–30]. Full-length MSP-2 (FC27 clone) expressed in *E. coli* was kindly provided by Robin Anders (La Trobe University, Australia). The schizont extract was prepared according to a previously published method [31]. Briefly, magnetic-activated cell sorting (MACS) purified schizont pellet was mixed with three times the volume of the pellet with PBS. Cell-lysis was done by freeze-thawing for 6 times, then it was spun down to clarify the supernatant and this extract was used after optimizing the coating concentration.

Each merozoite antigen was coated at 0.5–2 µg/ml and schizont extract at 1:8000 dilution on to 384 well NUNC MaxiSorp™ plates (Thermo Fisher Scientific Inc, MA, USA) and left overnight at 4 °C. The plates were washed with PBS/Tween 20 and non-specific binding was blocked with 0.1% casein (Thermo Fisher Scientific) on the following day. The plates were then incubated with participant sera diluted at 1:250 in 0.1% casein in triplicates for 1 h and washed. Horseradish peroxidase-conjugated goat anti-human IgG (Life Technologies, Australia) was added at 1:2500 dilution for 1 h and plates were again washed. ABTS [2,2′-azino-bis(3-ethylbenzthiazoline-6-sulfonic acid)] was added as enzyme–substrate for 15 min and absorbance at 405 nm was measured using a BMG POLARstar Omega fluorimeter (BMG Labtech, Germany).

### Measurement of total IgG levels against VSA

Total IgG antibody levels against VSAs expressed on the surface of IEs were measured by flow cytometry as previously described [32] with slight modifications. These antibodies are believed to primarily target *P. falciparum* erythrocyte membrane antigen 1 (PfEMP1) [33]. In brief, 2.5 μl of patient sera (1:20 dilution) were co-incubated with IEs at 0.2% haematocrit and at approximately 7–8% parasitaemia, diluted in PBS solution with 1% HI-FBS (Heat inactivated fetal bovine serum) for 30 min. Following incubation the cells were washed 3 times with PBS/1% HI-FBS and incubated with 25 μl of 1:100 rabbit anti-human IgG (Dako, Australia) diluted in PBS/1% HI-FBS. The cells were washed again in PBS/1% HI-FBS and incubated with Alexa Fluor 647 donkey anti-rabbit IgG in 1:500 dilution (Life Technologies, Australia) and 10 μg/ml ethidium bromide (EtBr) in PBS/1% HI-FBS for 30 min. Following incubation the cells were washed in PBS/1% HI-FBS and resuspended in ice-cold 2% paraformaldehyde fixative solution (prepared in PBS). The fixed IEs were then run through a HyperCyt^®^ system with a plate reader adapter (Intellicyt^®^, NM, USA) connected to a Cyan flow cytometer (Beckman Coulter Inc., CA, USA) where the cells were acquired. The flow cytometry data was analysed according to a previously published method [32].

### Data analysis

Statistical analyses were performed using Stata version 13.0 (StataCorp, Texas, USA). Statistical analyses were performed according to a pre-planned and approved analysis plan available at [34]. Measured antibody levels (in optical density [OD] for schizont and merozoite antigens and geometric mean fluorescence intensity [MFI] for VSA) were presented as a percentage of the positive control. The positive control came from a pool of plasma from malaria immune African adults whereas the negative control came from plasma samples from 3 Melbourne donors for the ELISA and 8 Melbourne donors for the flow cytometry assay.

Seroprevalence was defined as the percentage of the cohort having antibody responses greater than the mean antibody response plus three standard deviations for negative controls, which were malaria naïve samples from Melbourne blood donors. Socioeconomic status (SES) was calculated on the basis of a scoring system for household assets (HHA) adapted from [35].

Participant characteristics including demographic and basic clinical characteristics were categorized by intervention groups and the median and interquartile range for each characteristic were tabulated. Differences in characteristics across the groups were determined by Mann–Whitney test (non-parametric continuous variables with two groups), Kruskal–Wallis (non-parametric continuous variables with more than two groups) or Chi^2^ test (for categorical variables) where applicable. Differences in the antibody level and the seropositivity between 6 and 18 months old children were determined by Wilcoxon matched-pairs signed-ranks test and McNemar’s test, respectively. Statistical differences between the groups were reported as p < 0.05 and 95% confidence intervals were also reported for the analyses.

Antibody levels at 6 and 18 months of age were reported as the median percentage of the positive control and the interquartile range (IQR). The Kruskal–Wallis test was performed to compare antibody levels across supplementation groups. Regression analyses were carried out using natural logarithmically transformed antibody levels which were back transformed for reporting descriptive results. Linear regression univariate analysis was performed between LNS versus IFA, LNS versus MMN and MMN versus IFA to determine the antibody level differences between supplementation groups. Multivariate regression was also performed adjusting for the following covariates: maternal BMI at enrolment, duration of gestation (from enrolment to delivery), number of pregnancies, sex of the child, maternal education, proxy for SES, study site, maternal anaemic status at enrolment, maternal HIV status and bed net use by children and these covariates were uniformly included in all the adjusted analyses according to the pre-specified analysis plan. For both univariate and multivariate analyses, coefficients and 95% confidence intervals (CI) were reported. The number and the percentage of children who were seropositive for each malaria antigen were reported by supplementation groups, and chi^2^ test was performed to determine the differences across the supplementation groups. Univariate logistic regression was performed between LNS versus IFA, LNS versus MMN and MMN versus IFA to determine the differences in seropositivity between different supplementation groups. Multivariate logistic regression was performed adjusting for the above-mentioned covariates, reporting odds ratios (OR) and 95% CI.

## Results

### Study population characteristics

**Fig. 1:**
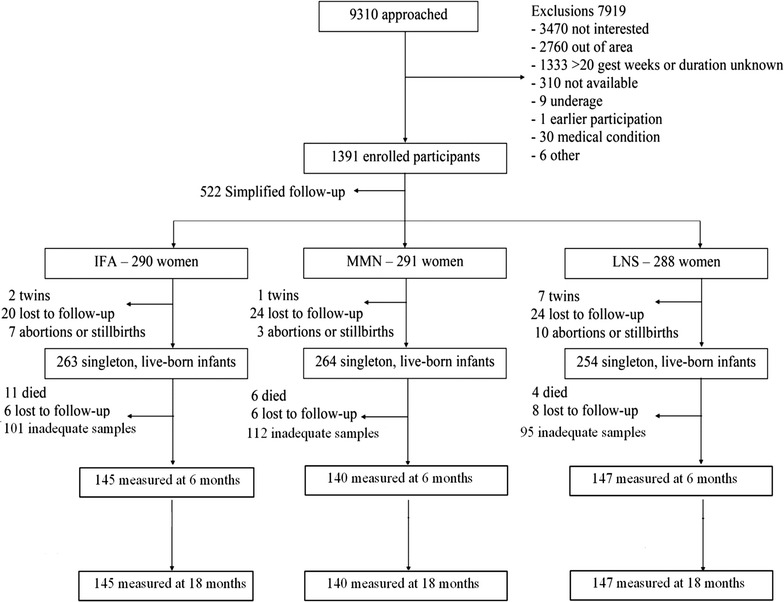
Participant flow in Consolidated Standards of Reporting Trials recommended format; adapted and modified from [22]. *gest* gestation, *IFA* iron and folic acid, *LNS* lipid-based nutrient supplement, *MMN* multiple micronutrients

From a total of 1391 enrolled pregnant women recruited to the trial, 869 completed the intervention and follow-up to 18 months after delivery. Four hundred and thirty-two singleton children from these women with sample availability at both 6 and 18 months were tested for this study (Fig. 1).

Of the 432 children (47.9% male and 52.1% female) tested at 6 and 18 months of age, 33.6% were from the IFA group, 32.4% from the MMN group and 34.0% in the LNS group. Table 1 summarizes the participant characteristics according to different supplementation groups. The characteristics did not differ substantially between the three intervention groups. The percentages of children who were parasitaemic at 6 months were higher in children from the IFA group (8.3% by microscopy and 7.6% by RDT) compared to MMN (3.6% by microscopy and 7.1% by RDT) or LNS (3.4% by microscopy and 6.8% by RDT) but the differences were non-significant.

**Table 1:** Participant characteristics according to supplementation groups

Characteristics	IFA^a^ n (%)	MMN^b^ n (%)	LNS^c^ n (%)	P value^d^
Number of children	145 (33.6)	140 (32.4)	147 (34.0)	0.87
Male	69 (47.6)	61 (43.6)	77 (52.4)	0.32
Parasitaemia by microscopy at 6 months	12 (8.3)	5 (3.6)	5 (3.4)	0.10
Parasitaemia by RDT^e^ at 6 months	11 (7.6)	10 (7.1)	10 (6.8)	0.97
Haemoglobin level at 6 months, mean ± SD, g/l	102.7 ± 16.7	103.6 ± 16.0	103.7 ± 14.7	0.74
Anaemia^f^ at 6 months	86 (59.3)	81 (57.9)	84 (57.1)	0.93
Low socioeconomic status	95 (65.5)	75 (53.6)	81 (55.1)	0.08
Mother’s education below median	79 (54.5)	66 (47.1)	80 (54.4)	0.34

^a^Iron and folic acid supplementation

^b^Multiple micronutrient supplementation

^c^Lipid-based nutrient supplements

^d^*P* value obtained by Kruskal–Wallis test (continuous variables) or Chi square test

^e^Rapid diagnostic test

^f^Anaemia defined as haemoglobin level < 110 g/l (World Health Organization [1])

The characteristics of the included and excluded children at 6 months are illustrated in Table 2. Significantly more included children had parasitaemia by microscopy (P = 0.01), but results of RDT showed no such difference. The percentage of anaemia at 6 months was significantly higher (P < 0.0001) in the excluded participants (71.7%) than the included group (58.1%).

**Table 2:** Comparative characteristics of the included and excluded participants at 6 months of age

Characteristics	Included (432)	Excluded (349)	P value^a^
Parasitaemia by microscopy	22 (5.1)	6 (1.7)	0.01
Parasitaemia by RDT^b^	31 (7.17)	35 (10.0)	0.15
Haemoglobin level, mean ± SD, g/l	103.4 ± 15.8	101.4 ± 15.9	0.09
Anaemia^c^	251 (58.1)	250 (71.7)	< 0.0001
Low socioeconomic status	251 (58.1)	215 (61.6)	0.32
Mother’s education below median	225 (52.1)	193 (55.3)	0.37

Values are number (%) or mean ± SD

^a^*P* value obtained by Chi square test or Mann–Whitney test (continuous variables)

^b^Rapid diagnostic test

^c^Anaemia defined as haemoglobin level < 110 g/l (World Health Organization [1])

### The magnitude and prevalence of antibodies in different age groups

The levels of antibodies and seroprevalence for the antibodies against merozoite antigens, schizont extract and VSA for three different parasite lines were measured at 6 months and 18 months of age (Table 3). The antibody levels to the merozoite antigens and schizont extract were significantly higher at 18 months compared to the levels at 6 months and it was statistically significant (P < 0.0001) for MSP1, MSP2 and EBA175.

**Table 3:** Magnitude and prevalence of antibodies in 432 children at 6 and 18 months

Antigen tested/Pf^a^ isolate	Antibody level^b^		P value^d^	Antibody seropositivity^c^ (%)		P value^e^
	6 months	18 months		6 months	18 months	
MSP1^f^	0.97 [0.38, 2.89]	2.80 [0.83, 6.35]	*<* *0.0001*	128 (29.6)	236 (54.6)	*<* *0.0001*
MSP2^g^	2.14 [1.31, 3.59]	2.95 [0.92, 9.12]	*<* *0.0001*	114 (26.3)	117 (27.0)	0.1004
EBA175^h^	2.50 [1.44, 4.13]	3.73 [2.19, 18.73]	*<* *0.0001*	48 (11.1)	51 (11.8)	0.5764
Rh2A9^i^	5.49 [2.01, 33.78]	8.5 [4.68, 14.29]	0.2829	14 (3.2)	122 (28.2)	*<* *0.0001*
Schizont	1.92 [0.84, 6.99]	3.32 [1.44, 6.89]	0.2522	202 (46.7)	233 (53.9)	*0.0314*
E8B	0.20 [0.03, 0.45]	0 [0, 0.21]	*<* *0.0001*	105 (25.2)	34 (7.8)	*<* *0.0001*
R29	0.18 [0, 0.63]	0 [0, 0.11]	*<* *0.0001*	37 (9.1)	16 (3.7)	*0.0016*
3D7	0.23 [0.01, 0.53]	0 [0, 0.29]	*<* *0.0001*	94 (22.2)	36 (8.3)	*<* *0.0001*

^a^ *Plasmodium falciparum*

^b^Antibody level presented as a percentage of the positive control showing the median and interquartile range

^c^Seropositivity defined as sample mean optical density or fluorescence intensity > mean + 3 standard deviations of the negative controls

^d^P value calculated using Wilcoxon matched-pairs signed-ranks test

^e^P value calculated using McNemar’s test

^f^Merozoite surface protein 1

^g^Merozoite surface protein 2

^h^Erythrocyte binding antigen 175

^i^Reticulocyte binding protein homologue 2A

Significant P values <0.05 are indicated in italics

However, the levels of naturally acquired IgG against VSAs were significantly lower in children at 18 months of age compared to the same children at 6 months. This difference was significant (< 0.0001) for all the tested parasite lines.

As with the antibody level data, seroprevalence of antibodies against the tested merozoite antigens and schizont extract were also significantly higher at 18 months compared to the seroprevalence at 6 months. The highest percentage of seropositivity was observed against MSP1 in 18 month old children; 54.6% compared to 29.6% seropositives at 6 months (P < 0.0001). Significant increases in seropositivity with increasing age were also observed for Rh2A9 (P < 0.0001) and schizont extract (P = 0.0314). However, the seroprevalence of IgG against VSAs of different parasite lines declined significantly between 6 and 18 months of age for the three tested parasite lines (P < 0.0001 for IgG against E8B and 3D7 and P = 0.0016 for R29).

### Association between nutrient supplementation and antibody seroprevalence in 6 months old children

Seroprevalence of the malaria antigens in 6 months old children was not significantly different between any of the treatment arms for any of the tested antigens except Rh2A9 (P = 0.044) (Table 4). Adjustment of the analysis for the selected covariates did not significantly change the results of the analysis, and the only significant difference was observed for the odds ratio of Rh2A9 between LNS and IFA group in the univariate analysis (P = 0.047), with the odds of being seropositive at 6 months of age in LNS group being 54% less than in the IFA group. This association did not remain significant in the adjusted analysis.

**Table 4:** Association between nutrient supplementation and seroprevalence in 6 months old children

Outcome	Number of children seropositive/total number of children				Comparison between LNS and IFA group		Comparison between LNS and MMN group		Comparison between MMN and IFA group	
	IFA^a^	MMN^b^	LNS^c^	P value^d^	OR (95% CI)	P value^e^	OR (95% CI)	P value^e^	OR (95% CI)	P value^e^
MSP-1^f^ 19kD	43/145 (29.7%)	44/140 (31.4%)	41/147 (27.9%)	0.806	0.96 (0.74, 1.23)	0.739	0.84 (0.51, 1.40)	0.512	1.07 (0.64, 1.77)	0.804
Adjusted model^g^					0.97 (0.73, 1.28)	0.808	0.86 (0.49, 1.49)	0.583	1.01 (0.57, 1.76)	0.981
MSP-2^h^	38/145 (26.2%)	40/140 (28.6%)	36/147 (24.5%)	0.734	0.96 (0.73, 1.24)	0.736	0.81 (0.48, 1.37)	0.434	1.07 (0.63, 1.80)	0.807
Adjusted model^g^					0.98 (0.74, 1.31)	0.893	0.80 (0.45, 1.42)	0.447	1.09 (0.62, 1.94)	0.764
EBA-175^i^	15/145 (10.3%)	20/140 (14.3%)	13/147 (8.9%)	0.320	0.92 (0.62, 1.35)	0.663	0.58 (0.28, 1.22)	0.152	1.34 (0.65, 2.76)	0.426
Adjusted model^g^					0.96 (0.63, 1.47)	0.857	0.54 (0.25, 1.18)	0.120	1.53 (0.70, 3.37)	0.289
Rh2A9^j^	9/145 (6.21%)	3/140 (2.14%)	2/147 (1.4%)	*0.044*	0.46 (0.21, 0.99)	*0.047*	0.63 (0.10, 3.83)	0.616	0.33 (0.09, 1.23)	0.098
Adjusted model^g^					0.48 (0.21, 1.06)	0.070	0.79 (0.12, 5.42)	0.814	0.34 (0.09, 1.30)	0.114
Schizont extract	68/145 (46.9%)	67/140 (47.86%)	67/147 (45.6%)	0.927	0.97 (0.77, 1.23)	0.821	0.91 (0.57, 1.45)	0.699	1.07 (0.67, 1.71)	0.774
Adjusted model^g^					0.91 (0.70, 1.18)	0.468	0.89 (0.53, 1.51)	0.677	0.97 (0.58, 1.63)	0.923
VSA^k^ of E8B parasite line	31/145 (21.4%)	39/140 (27.9%)	35/147 (23.8%)	0.437	1.09 (0.83, 1.43)	0.552	0.80 (0.47, 1.36)	0.402	1.52 (0.88, 2.64)	0.134
Adjusted model^g^					1.01 (0.74, 1.37)	0.957	0.70 (0.38, 1.26)	0.233	1.55 (0.86, 2.80)	0.146
VSA of R29 parasite line	14/145 (9.7%)	12/140 (8.6%)	11/147 (7.5%)	0.803	0.86 (0.57, 1.30)	0.470	0.83 (0.35, 1.94)	0.659	0.88 (0.39, 1.98)	0.756
Adjusted model^g^					0.76 (0.48, 1.20)	0.234	0.69 (0.27, 1.74)	0.427	0.91 (0.38, 2.14)	0.822
VSA of 3D7 parasite line	27/145 (18.6%)	33/140 (23.6%)	34/147 (23.1%)	0.530	1.16 (0.87, 1.54)	0.313	0.97 (0.56, 1.69)	0.924	1.35 (0.76, 2.41)	0.301
Adjusted model^g^					1.04 (0.75, 1.43)	0.829	0.89 (0.48, 1.65)	0.721	1.24 (0.66, 2.31)	0.501

^a^Iron and folic acid

^b^Multiple micronutrients

^c^lipid based nutrient supplements

^d^ P value calculated using the Chi^2^ test

^e^ P value calculated using logistic regression reporting Odds Ratios (OR) and 95% Confidence intervals (CI)

^f^Merozoite surface protein 1

^g^P value calculated using multivariate logistic regression reporting odds ratios (OR) while adjusting for maternal BMI at enrolment, duration of gestation (from enrolment to delivery), number of pregnancies, sex of the child, maternal education, socioeconomic status, study site, maternal anaemic status at enrolment, maternal HIV status and bed net use by children

^h^Merozoite surface protein 2

^i^Erythrocyte binding antigen 175

^j^Reticulocyte binding protein homologue 2A

^k^Variant surface antigens

Significant P values <0.05 are indicated in italics

### Association between nutrient supplementation and antibody seroprevalence in 18 months old children

Seroprevalence of the tested malaria antigens in 18 months old children was not significantly different between any of the treatment arms for any of the antigens tested (Table 5). Adjustment of the analysis for the selected covariates did not significantly change the results of the analysis, and no significant differences in antibody seroprevalence were observed between the different supplementation groups for any of tested antigens.

**Table 5:** Association between nutrient supplementation and seroprevalence in 18 months old children

Outcome	Number of children seropositive/total number of children				Comparison between LNS and IFA group		Comparison between LNS and MMN group		Comparison between MMN and IFA group	
	IFA^a^	MMN^b^	LNS^c^	P value^d^	OR (95% CI)	P value^e^	OR (95% CI)	P value^e^	OR (95% CI)	P value^e^
MSP-1 19kD^f^	82/145 (56.5%)	72/140 (51.4%)	82/147 (55.7%)	0.646	0.98 (0.78, 1.24)	0.895	1.19 (0.75, 1.90)	0.460	0.83 (0.52, 1.33)	0.444
Adjusted model^g^					0.98 (0.77, 1.25)	0.865	1.10 (0.67, 1.82)	0.708	0.80 (0.48, 1.34)	0.398
MSP-2^h^	42/145 (28.9%)	39/140 (27.8%)	36/147 (24.4%)	0.669	0.88 (0.66, 1.16)	0.359	0.79 (0.45, 1.40)	0.428	0.95 (0.54, 1.66)	0.851
Adjusted model^g^					0.82 (0.60, 1.13)	0.226	0.63 (0.34, 1.18)	0.149	0.96 (0.53, 1.76)	0.906
EBA-175^i^	19/145 (13.1%)	14/140 (10.0%)	18/147 (12.2%)	0.705	0.99 (0.67, 1.43)	0.967	1.59 (0.69, 3.63)	0.273	0.61 (0.27, 1.40)	0.242
Adjusted model^g^					0.91 (0.60, 1.37)	0.642	1.19 (0.48, 2.95)	0.710	0.65 (0.27, 1.58)	0.342
Rh2A9^j^	42/145 (28.9%)	40/140 (28.5%)	40/147 (27.2%)	0.941	0.96 (0.74, 1.24)	0.739	0.93 (0.56, 1.57)	0.797	0.96 (0.57, 1.61)	0.880
Adjusted model^g^					0.96 (0.73, 1.26)	0.763	0.84 (0.48, 1.47)	0.540	0.98 (0.56, 1.71)	0.931
Schizont extract	73/145 (50.3%)	78/140 (55.7%)	82/147 (55.7%)	0.568	1.12 (0.89, 1.40)	0.352	1.003 (0.63, 1.60)	0.991	1.24 (0.78, 1.99)	0.365
Adjusted model^g^					1.18 (0.90, 1.54)	0.223	0.87 (0.52, 1.46)	0.600	1.40 (0.83, 2.35)	0.208
VSA^k^ of E8B parasite line	14/145 (9.6%)	11/140 (7.9%)	9/147 (6.1%)	0.534	0.78 (0.51, 1.21)	0.266	0.76 (0.30, 1.89)	0.554	0.78 (0.33, 1.83)	0.562
Adjusted model^g^					0.80 (0.50, 1.29)	0.360	0.72 (0.27, 1.89)	0.504	0.75 (0.29, 1.93)	0.557
VSA of R29 parasite line	7/145 (4.8%)	5/140 (3.5%)	4/147 (2.7%)	0.632	0.74 (0.40, 1.39)	0.351	0.76 (0.20, 2.87)	0.680	0.72 (0.22, 2.32)	0.582
Adjusted model^g^					0.79 (0.39, 1.60)	0.506	0.95 (0.22, 4.07)	0.946	0.85 (0.23, 3.14)	0.808
VSA of 3D7 parasite line	15/145 (10.3%)	11/140 (7.8%)	10/147 (6.8%)	0.532	0.80 (0.52, 1.21)	0.283	0.86 (0.35, 2.08)	0.732	0.85 (0.37, 1.97)	0.710
Adjusted model^g^					0.74 (0.46, 1.22)	0.241	0.55 (0.20, 1.52)	0.250	1.11(0.43, 2.83)	0.830

^a^Iron and folic acid

^b^Multiple micronutrients

^c^lipid based nutrient supplements

^d^ P-value calculated using the Chi^2^ test

^e^ P-value calculated using logistic regression reporting Odds Ratios (OR) and 95% Confidence intervals (CI)

^f^Merozoite surface protein 1

^g^P-value calculated using multivariate logistic regression reporting odds ratios (OR) while adjusting for maternal BMI at enrolment, duration of gestation (from enrolment to delivery), number of pregnancies, sex of the child, maternal education, socioeconomic status, study site, maternal anaemic status at enrolment, maternal HIV status and bed net use by children

^h^Merozoite surface protein 2

^i^Erythrocyte binding antigen 175

^j^Reticulocyte binding protein homologue 2A

^k^Variant surface antigens

### Association between nutrient supplementation and antibody levels in 6 month old children

The level of antibodies did not differ significantly according to different nutrient supplementation groups for any of the tested antigens at 6 months of age. Moreover, multivariate linear regression showed no significant differences in the levels of antibodies against any of the tested antigens when they were categorized by different supplementation groups (Table 6).

**Table 6:** Association between nutrient supplementation and antibody levels in 6 months old children

Outcome	Antibody levels by study group, median (IQR)				LNS and IFA		LNS and MMN		MMN and IFA	
	IFA^a^	MMN^b^	LNS^c^	P value^d^	Coeff (95% CI)	P value^e^	Coeff (95% CI)	P value^e^	Coeff (95% CI)	P value^e^
Number of participants	N = 145	N = 140	N = 147							
MSP-1 19kD^f^	0.81 (0.38, 2.45)	1.13 (0.37, 3.02)	1.03 (0.40, 0.72)	0.430	1.03 (0.85, 1.25)	0.773	0.88 (0.60, 1.28)	0.498	1.15 (0.78, 1.70)	0.467
Adjusted model^g^					1.06 (0.87, 1.30)	0.542	0.94 (0.63, 1.39)	0.755	1.11 (0.75, 1.64)	0.614
MSP-2^h^	2.36 (1.43, 3.57)	2.02 (1.33, 4.13)	1.98 (1.19, 3.27)	0.313	0.99 (0.87, 1.12)	0.833	0.87 (0.66, 1.15)	0.334	1.08 (0.82, 1.42)	0.592
Adjusted model^g^					0.99 (0.86, 1.13)	0.834	0.88 (0.66, 1.19)	0.417	1.06 (0.80, 1.40)	0.703
EBA-175^i^	2.37 (1.34, 3.85)	2.60 (1.50, 4.50)	2.50 (1.42, 4.02)	0.335	1.03 (0.92, 1.14)	0.610	0.91 (0.74, 1.13)	0.390	1.14 (0.91, 1.43)	0.240
Adjusted model^g^					1.04 (0.93, 1.16)	0.502	0.86 (0.69, 1.08)	0.195	1.24 (0.99, 1.54)	0.061
Rh2A9^j^	4.93 (1.53,18.55)	5.97 (2.12, 39.95)	5.86 (2.45, 33.70)	0.245	1.11 (0.89, 1.38)	0.364	1.00 (0.66, 1.53)	0.986	1.23 (0.77, 1.95)	0.382
Adjusted model^g^					1.08 (0.85, 1.37)	0.532	0.99 (0.63, 1.57)	0.990	1.16 (0.70, 1.93)	0.559
Schizont extract	1.85 (0.80, 4.93)	2.11 (0.82, 8.08)	2.06 (0.89, 7.23)	0.569	1.07 (0.89, 1.28)	0.465	0.97 (0.68, 1.40)	0.875	1.21 (0.85, 1.73)	0.282
Adjusted model^g^					1.06 (0.89, 1.27)	0.481	0.88 (0.61, 1.26)	0.475	1.30 (0.91, 1.85)	0.155
VSA^k^ of E8B parasite line	0.17 (0.03, 0.46)	0.26 (0.06, 0.47)	0.19 (0.02, 0.44)	0.453	1.04 (0.87, 1.24)	0.688	1.00 (0.70, 1.42)	0.995	1.07 (0.76, 1.51)	0.709
Adjusted model^g^					1.00 (0.82, 1.21)	0.996	0.99 (0.68, 1.47)	0.987	1.02 (0.71, 1.47)	0.925
VSA of R29 parasite line	0.16 (0, 0.59)	0.25 (0, 0.81)	0.12 (0, 0.55)	0.452	0.93 (0.75, 1.15)	0.496	0.70 (0.46, 1.07)	0.098	1.21 (0.80, 1.82)	0.358
Adjusted model^g^					0.91 (0.73, 1.13)	0.407	0.63 (0.41, 0.97)	0.063	1.30 (0.88, 1.91)	0.187
VSA of 3D7 parasite line	0.19 (0.01, 0.48)	0.24 (0.02, 0.64)	0.26 (0.01,0.59)	0.544	1.19 (0.98, 1.46)	0.081	1.06 (0.72, 1.55)	0.774	1.34 (0.91, 1.99)	0.138
Adjusted^g^					1.13 (0.92, 1.39)	0.245	1.05 (0.69, 1.61)	0.810	1.18 (0.81, 1.73)	0.382

^a^Iron and folic acid

^b^Multiple micronutrients

^c^lipid based nutrient supplements

^d^ P value calculated using Kruskal–Wallis test

^e^ P value calculated using linear regression of antibody levels between supplementation groups reporting coefficient and 95% Confidence intervals (CI)

^f^Merozoite surface protein 1

^g^P value calculated using multivariate regression adjusting for maternal BMI at enrolment, duration of gestation (from enrolment to delivery), number of pregnancies, sex of the child, maternal education, socioeconomic status, study site, maternal anaemic status at enrolment, maternal HIV status and bed net use by children

^h^Merozoite surface protein 2

^i^Erythrocyte binding antigen 175

^j^Reticulocyte binding protein homologue 2A

^k^Variant surface antigens

### Association between nutrient supplementation and antibody levels in 18 month old children

The level of antibodies did not differ significantly between different nutrient supplementation groups for any of the tested antigens except for IgG against E8B parasite line (P = 0.043) in 18 months old children. Multivariate linear regression showed no significant differences in the levels of antibodies against any of the tested antigens between the different supplementation groups (Table 7).

**Table 7:** Association between nutrient supplementation and antibody levels in 18 months old children

Outcome	Antibody levels by study group, median (IQR)				LNS and IFA		LNS and MMN		MMN and IFA	
	IFA^a^	MMN^b^	LNS^c^	P value^d^	Coeff (95% CI)	P value^e^	Coeff (95% CI)	P value^e^	Coeff (95% CI)	P value^e^
Number of participants	N = 145	N = 140	N = 147							
MSP-1 19kD^f^	2.59 (0.83, 6.09)	2.95 (0.75, 6.37)	2.64 (1.00, 6.59)	0.877	1.05 (0.87, 1.26)	0.603	1.02 (0.70, 1.49)	0.909	1.09 (0.75, 1.58)	0.663
Adjusted model^g^					1.04 (0.86, 1.26)	0.681	0.97 (0.65, 1.42)	0.857	1.07 (0.72, 1.58)	0.745
MSP-2^h^	3.10 (0.97, 9.81)	2.74 (0.64, 8.57)	3.21 (1.38, 9.05)	0.334	1.01 (0.83, 1.22)	0.938	1.12 (0.77, 1.64)	0.548	0.92 (0.61, 1.38)	0.683
Adjusted model^g^					0.99 (0.82, 1.22)	0.984	1.17 (0.79, 1.74)	0.434	0.86 (0.56, 1.31)	0.477
EBA-175^i^	4.06 (2.32, 19.29)	3.39 (1.88, 20.11)	3.62 (2.29, 18.47)	0.425	0.94 (0.80, 1.10)	0.427	0.99 (0.07, 1.38)	0.973	0.89 (0.64, 1.24)	0.489
Adjusted model^g^					0.90 (0.77, 1.06)	0.217	0.98 (0.70, 1.36)	0.894	0.84 (0.60, 1.17)	0.294
Rh2A9^j^	8.96 (4.66,13.50)	8.38 (4.77, 14.46)	8.76 (4.86,15.17)	0.952	1.02 (0.92, 1.12)	0.723	1.02 (0.85, 1.22)	0.848	1.02 (0.83, 1.24)	0.869
Adjusted model^g^					1.03 (0.93, 1.14)	0.566	1.03 (0.84, 1.25)	0.790	1.05 (0.85, 1.29)	0.679
Schizont extract	3.13 (1.69, 6.19)	3.94 (1.40, 8.18)	2.96 (1.28, 6.35)	0.407	0.94 (0.80, 1.10)	0.433	0.79 (0.56, 1.12)	0.185	1.10 (0.80, 1.49)	0.564
Adjusted model^g^					0.91 (0.78, 1.09)	0.332	0.78 (0.54, 1.12)	0.179	1.10 (0.80, 1.51)	0.559
VSA^k^ of E8B parasite line	0.00 (0.00, 0.27)	0.00 (0.00, 0.25)	0.00 (0.00, 0.12)	*0.043*	0.82 (0.60, 1.11)	0.189	0.62 (0.35, 1.10)	0.099	0.99 (0.60, 1.61)	0.953
Adjusted model^g^					0.83 (0.61, 1.14)	0.250	0.61 (0.34, 1.11)	0.102	0.95 (0.60, 1.51)	0.818
VSA of R29 parasite line	0.00 (0.00, 0.07)	0.00 (0.00, 0.24)	0.00 (0.00, 0.004)	0.223	0.86 (0.58, 1.29)	0.469	0.89 (0.39, 1.98)	0.756	0.85 (0.39, 1.84)	0.671
Adjusted model^g^					0.82 (0.53, 1.28)	0.382	0.73 (0.32, 1.70)	0.465	0.95 (0.43, 2.06)	0.889
VSA of 3D7 parasite line	0.002 (0.00, 0.39)	0.00 (0.00, 0.24)	0.00 (0.00, 0.28)	0.150	1.05 (0.78, 1.43)	0.731	1.18 (0.64, 2.19)	0.592	0.98 (0.56, 1.71)	0.930
Adjusted^g^					1.14 (0.82, 1.59)	0.441	1.11 (0.55, 2.25)	0.765	0.94 (0.50, 1.75)	0.834

^a^Iron and folic acid

^b^Multiple micronutrients

^c^lipid based nutrient supplements

^d^ P value calculated using Kruskal–Wallis test

^e^ P value calculated using linear regression of antibody levels between supplementation groups reporting coefficient and 95% Confidence intervals (CI)

^f^Merozoite surface protein 1

^g^P value calculated using multivariate regression adjusting for maternal BMI at enrolment, duration of gestation (from enrolment to delivery), number of pregnancies, sex of the child, maternal education, socioeconomic status, study site, maternal anaemic status at enrolment, maternal HIV status and bed net use by children

^h^Merozoite surface protein 2

^i^Erythrocyte binding antigen 175

^j^Reticulocyte binding protein homologue 2A

^k^Variant surface antigens

Significant P values <0.05 are indicated in italics

## Discussion

This study investigated the impact of nutritional supplementation on malarial immunity in a subset of young children from a randomized controlled trial of pre-natal nutrient supplementation with IFA or pre- and postnatal MMN or LNS. Antibodies to several important merozoite antigens (MSP1, MSP2, EBA175, Rh2A9), schizont extract and VSA for three different parasite lines (E8B-ICAM, R29 and 3D7 overexpressing *var* A) were measured in order to determine whether nutrient supplementation improves the acquisition of malaria antibody in young children.

In malaria endemic areas, antibodies to malaria antigens increase with age, being higher in adults than in children, but the dynamics of antibody production in infants are less well studied [36]. The observations that the levels and seroprevalence of antibody to most of the merozoite antigens tested increased between 6 and 18 months of age are in agreement with observations in a case control study conducted in Kenyan children, in which the level of antibodies against MSP1 and PfRh2 increased steadily with increasing age from birth to 2 years [37], and a cohort study from Benin [38], in which MSP1 and MSP2 antibody levels showed a constant increase until 18 months of age. In a higher transmission setting, the seroprevalence of antibody to MSP1 reached a peak after 6 months of age, whereas prevalence of antibody to MSP2 peaked at 9 months of age [39].

In contrast to the increase in antibodies to merozoite antigens, the levels of antibodies against VSA decreased from 6 to 18 months, suggesting an ongoing loss of maternal anti-VSA antibodies with little or no development of the child’s own VSA antibody responses. Consistent with this, in a recent study anti-VSA antibodies waned by 6–9 months of age and did not reappear during infancy and early childhood [40]. It is possible that the anti-VSA antibodies require more infections than the merozoite antigens to develop, and anti-VSA responses are known to often be short-lived following infection [41]. This may be related to the highly variant nature of VSA [42], requiring repeated exposure to develop cross-reactive antibodies to multiple VSA variants, whereas merozoite antigens are more conserved.

The study found no evidence that nutrient supplementation altered the development of antibody responses to either merozoite antigens or VSA in young children, in accordance with a study conducted on Beninese children of similar age group in whom there was no association between nutritional intake and anti-malarial antibody levels [38]. Antibody levels or seroprevalence of antibodies to the tested antigens did not differ between the supplementation groups in either unadjusted or adjusted analysis (P > 0.05) except for the seroprevalence in 6 months old children for Rh2A9 (P = 0.044), and that significance was lost in the adjusted analysis. The trial did not demonstrate differences in growth or morbidity events between the different supplementation arms and suggested that the nutritional supplements provided were not sufficient by themselves to promote infant growth [22]. This may also explain why no differences were seen in anti-malaria antibody levels between the intervention groups.

Clinical studies have suggested that under-nutrition has an impact on early childhood mortality and morbidity [2, 43]. However, other studies did not find any relationship between protein energy malnutrition or stunting and clinical malaria occurrence [7, 44]. The interaction between nutrition and malarial immunity has been relatively little studied. Among Senegalese pre-school children, those children who were stunted had lower levels of IgG to schizont extract compared to non-stunted children [45], and Papua New Guinean children with wasting were reported to have lower levels of specific IgG to blood stage malaria antigens [4]. But these previous studies of the impact of nutritional status on malarial immunity were observational and therefore subjected to confounding. The only statistically significant difference between the supplement groups seen in the present study in 6 months old children was the seroprevalence against Rh2A9 between three treatment arms (P = 0.044); the univariate analysis also showed significant differences in the odds ratio between LNS and IFA group. There was no significant association in the adjusted analysis. Although IgG against VSA in E8B parasite line in 18 months old children was significantly different in unadjusted analyses between groups (P = 0.043), this, together with the Rh2A9 observations, were most probably chance findings, in the absence of similar findings in other assays.

Strengths of the study include the substantial sample size of children from a randomized controlled trial with paired samples at 6 and 18 months of age and the number of different antibody measurements performed. A high proportion of iLiNS study participants were stunted at 18 months of age, indicating a high prevalence of nutritional deprivation, frequent infection or inflammation in infants. Possible study weaknesses include the relatively low prevalence of severe nutritional deficits in the mothers [21] as a previous study showed that with protein energy supplementation, improved birth outcome was more prominently observed in malnourished mothers than marginally nourished or well-nourished mothers [46]. Other limitations could be the inclusion of only a subset of all children followed in the parent study [21], the potential selection bias due to differences in the included and excluded children, different assay platforms for measuring merozoite immunity (ELISA) compared to VSA immunity (flow cytometry), and the lack of functional assays of malaria immunity [36].

## Conclusions

In conclusion, this study reports the acquisition of malaria antibody in young children with different pre- and postnatal nutrient supplements and shows no significant changes in antibody acquisition among different supplement groups. It addresses the knowledge gap regarding the effect of nutrient supplementation on malarial immunity in young children. Further studies may be required to determine whether malaria immunity is significantly impaired in groups with severe nutritional depletion, or whether more substantial protein energy supplementation might help the development of malaria immunity, especially in high risk groups.

## Abbreviations

EBA: erythrocyte binding antigen
HHA: household assets
IE: infected erythrocytes
IFA: iron and folic acid
iLiNS: International Lipid-based Nutrient Supplement
IPTp: intermittent preventive treatment in pregnancy
IQR: interquartile range
ITN: insecticide-treated net
LNS: lipid-rich nutrient supplement
MACS: magnetic-activated cell sorting
MMN: multiple micronutrients
MSP: merozoite surface protein
OR: odds ratio
PCR: polymerase chain reaction
PfEMP1: *P. falciparum* erythrocyte membrane protein-1
PfRh2: *Plasmodium falciparum* reticulocyte binding homologue 2
Rh2A9: reticulocyte binding protein homologue 2A
SES: socioeconomic status
VSA: variant surface antigen
